# Music emotion recognition based on temporal convolutional attention network using EEG

**DOI:** 10.3389/fnhum.2024.1324897

**Published:** 2024-03-28

**Authors:** Yinghao Qiao, Jiajia Mu, Jialan Xie, Binghui Hu, Guangyuan Liu

**Affiliations:** ^1^School of Electronic and Information Engineering, Southwest University, Chongqing, China; ^2^Institute of Affective Computing and Information Processing, Southwest University, Chongqing, China; ^3^Chongqing Key Laboratory of Nonlinear Circuits and Intelligent Information Processing, Southwest University, Chongqing, China

**Keywords:** EEG, music emotion recognition, CNN, BiLSTM, self-attention

## Abstract

Music is one of the primary ways to evoke human emotions. However, the feeling of music is subjective, making it difficult to determine which emotions music triggers in a given individual. In order to correctly identify emotional problems caused by different types of music, we first created an electroencephalogram (EEG) data set stimulated by four different types of music (fear, happiness, calm, and sadness). Secondly, the differential entropy features of EEG were extracted, and then the emotion recognition model CNN-SA-BiLSTM was established to extract the temporal features of EEG, and the recognition performance of the model was improved by using the global perception ability of the self-attention mechanism. The effectiveness of the model was further verified by the ablation experiment. The classification accuracy of this method in the valence and arousal dimensions is 93.45% and 96.36%, respectively. By applying our method to a publicly available EEG dataset DEAP, we evaluated the generalization and reliability of our method. In addition, we further investigate the effects of different EEG bands and multi-band combinations on music emotion recognition, and the results confirm relevant neuroscience studies. Compared with other representative music emotion recognition works, this method has better classification performance, and provides a promising framework for the future research of emotion recognition system based on brain computer interface.

## 1 Introduction

Music, recognized as a conduit for emotional expression, possessed a formidable capacity to evoke a range of affective reactions in its listeners, including but not limited to joy, exhilaration, and apprehension (Day et al., 2009). In today’s society, music has been used as the best therapeutic tool (Raglio, 2023). Subsequent research has revealed that emotions play a pivotal role in shaping individuals’ music selection, while music itself serves as a vehicle for the expression of one’s emotional states (Konečni, 2003). Music has been the focus of research across various disciplines, including musicology, psychology, signal processing, and more, due to its ability to effectively convey emotions and elicit emotional reactions from listeners. Neurological studies have demonstrated that music serves as a valuable tool for evaluating brain systems (Peretz and Zatorre, 2005).

Based on the findings by Juslin et al. (2008) approximately 64% of musical experiences had an emotional impact on individuals, evoking feelings of happiness, joy, nostalgia, or longing. Additionally, a study conducted among young individuals demonstrated that listening to music is considered one of the most effective coping strategies for managing stress (Strasser et al., 2022). Sareen et al. (2020) studied intellectual development disorders by comparing the electroencephalogram (EEG) signals of the subjects in the resting state and the music state. Consequently, with the advent of the Internet era and the proliferation of multimedia applications, there was a growing emphasis on the significance of emotion-based music recommendation systems (Lampropoulos et al., 2012). Computing to estimate, interpret and process human emotions was an area of machine learning that was also used in areas as diverse as health, safety and education. In recent years, emotion recognition based on EEG received extensive attention in the field of human-computer interaction (Hsu et al., 2018; Nawaz et al., 2020; Naser and Saha, 2021).

Additionally, the field of human emotions encompasses several pivotal definitions and theories that provide valuable insights into this fundamental aspect of human psychology. According to Yorozu et al. (1987), emotional experience could be understood as a reaction to physiological alterations that occurred within the body. Therefore, it is important for emotions to understand the physiological response of each emotion. Russell (1980) proposed a common hypothesis that emotions consisted of two arousal and valence elements. Arousal indicated the level of emotional activation, while valence indicated positive or negative. This hypothesis systematically describes emotions and has been widely used as background knowledge in countless studies. Gong et al. (2023) used the DEAP dataset to classify valence and arousal in subjects. The average accuracy of valence and arousal were 82.75% and 84.22%, respectively (Gong et al., 2023). Zhou et al. (2021) collected EEG data from 40 participants to regulate negative emotions and calculated the binary predictions of arousal and valence (high or low) as 78.75% ± 9.48% and 73.98% ± 5.54%, respectively, using machine learning methods. In the aforementioned studies, valence and arousal are commonly employed to characterize emotional states. Thus, this study also employs the classification of valence and arousal states to recognize music-induced emotional states.

For an effective emotion recognition system, two crucial conditions must be fulfilled: high recognition accuracy and robust adaptability to diverse structures. By satisfying conditions, a dependable emotion recognition system can be formulated. In recent years, people have done a lot of research on emotion recognition system, these researches can be generally divided into three categories. The first type is the analysis of facial expression or the dialogue of characters, through the change of people’s facial expression and the content of the dialogue tone to identify emotions. The second category involves the identification of peripheral physiological signals associated with various emotional states, including electrocardiogram, electromyography, respiration, and pulse. Compared with the analysis of facial expressions and conversations, the assessment of peripheral physiological signals offers a more nuanced and informative approach to predict and recognize emotions by providing additional intricate details and valuable information (Shu et al., 2018). The third approach centers on analyzing brain signals generated by the central nervous system, such as EEG (Tagluk and Isik, 2019). The high temporal resolution, non-invasive nature, portability, and relatively low data processing cost make EEG a suitable option for investigating the neural associations of diverse cognitive functions, including emotion. In recent years, numerous studies have utilized EEG for emotion recognition. These studies have devised diverse computational methods utilizing EEG signals to facilitate automated observation and analysis of emotion recognition, which will be further examined in the following discussion. Traditional EEG emotion classification algorithms mainly include support vector machine (SVM) (Vapnik, 1963), *K*-nearest neighbor (KNN) (Cover and Hart, 1967), Random Forest (RF) (Breiman, 2001), and so on. However, these algorithms are unable to extract deeper emotional features, which may result in lower accuracy of emotion recognition. In recent years, there has been an increasing trend among researchers to employ deep learning models for emotion recognition. Numerous studies have utilized a combination of convolution neural network (CNN) and long short-term memory (LSTM) models to extract both temporal and spatial features for improved performance. Salama et al. (2018) used the CNN model for emotion recognition. The researchers used the DEAP dataset. Their CNN model reportedly achieved a final classification accuracy of 87.44% and 88.49% for valence and arousal, respectively. Keelawat et al. (2019) employed CNN to extract features from EEG signals for arousal and valence classification in 12 subjects. A comparison was made between the CNN and SVM for emotion recognition. The results revealed the superior performance of CNN over SVM, underscoring CNN’s efficacy in the field of emotion recognition. Jiang et al. (2021b) introduced a WT-CNN model that utilized wavelet transform to decompose EEG signals into multiple frequency bands, each containing emotional features. Subsequently, the decomposed signals were fed into a CNN to capture deep characteristics, achieving an accuracy of 80.65%. Given the temporal nature of EEG signals, researchers must also understand their temporal information. LSTM has emerged as a proficient model for analyzing time series data, making it a suitable choice for handling EEG signals. Yang et al. (2018) used a hybrid neural network that combines CNN and recursive neural networks (RNNs) to automatically recognize emotions from EEG signals. Liang et al. (2021) integrated CNN, RNN, and GAN networks to conduct unsupervised emotion recognition in DEAP, MAHNOB-HCI, and SEED open data sets. Ozdemir et al. (2021) presented a robust approach utilizing the CNN-LSTM model for emotion classification. EEG signals were transformed into topologies based on electrode positions, trained using CNN, and then time features were extracted from subsequent time Windows using LSTM, the recognition rate of arousal reached 86.13%, and the recognition rate of valence reached 90.62%. Despite the advancements made in the research of hybrid CNN-LSTM models, several challenges remain to be addressed.

For example, CNN’s convolution kernel can be perceived locally, but it may break these relationships. In the process of model training, one-way LSTM can only learn the past time information, but cannot obtain the future information of signal. And fully integrating the past and future information of signals can better identify emotions. We understand that Transformer (Vaswani et al., 2017) had a strong global awareness due to its self-attention mechanism (Devlin et al., 2018). BiLSTM, compared to LSTM, could effectively integrate both past and future information of the signal, enabling better handling of long sequences and long-range dependencies, thereby improving predictive performance (Huang et al., 2015).

Therefore, we proposed a new emotion recognition model based on CNN-LSTM, namely CNN-SA-BiLSTM (CSBN) model. The model utilized the advantages of CNN model in capturing local features, the advantages of self-attention mechanism in capturing global features, and the advantages of BiLSTM in extracting temporal features for emotion recognition. The model realized the recognition of music-induced emotional states and validated the effectiveness of the method by comparing it with machine learning and deep learning models. We also conducted experiments on DEAP, a widely used public data set. The experimental results showed that the model not only had a high emotion recognition accuracy, but also had a good robustness, which provided a design idea and research model for the design of emotion-based music recommendation system.

The contribution of this article can be summarized as follows:

1. The EEG database induced by musical stimuli (SWU-M) is designed, which can be used to classify emotions for specific topics.
2. Based on the CNN-LSTM model, combined with the advantages of self-attention mechanism in feature extraction and the feature recognition ability of BiLSTM, a novel emotion recognition method based on CSBN was proposed to classify emotion valence and arousal. The results show that this model is superior to the existing methods in terms of valence and arousal classification.
3. The influence of θ, α, β, and γ bands on music emotion recognition and the influence of multi-band combination on music emotion recognition were studied. The study showed that musical stimulation had the most obvious effect on the alpha band.

The rest of this article is organized as follows. In section “2 Materials and experimental instructions” we describe the Self-collecting Music Database (SWU-M), a set of EEG data collected through musical stimuli of four different emotions. Section “3 Proposed method” describes the proposed emotion recognition model framework, then preprocesses the collected EEG data, and finally describes the CSBN model architecture in detail. Section “4 Results” discusses extensive experiments to demonstrate the validity of the proposed CSBN. Section “5 Discussion” discusses the segmentation band recognition of EEG signals under music stimulation, and discusses the influence of music stimulation on music emotion recognition under different frequency bands and different frequency band combinations. Finally, the conclusion and future work are discussed in section “6 Conclusion and future work.”

## 2 Materials and experimental instructions

In this section, we describe the auditory stimulation-based EEG dataset (SWU-M), and the materials and procedures required for the experiment. The Ethical Review Committee of Southwest University approved the study protocol, and all methods were carried out within the committee guidelines (IRB No. SEIE2022091101). All participants received payment for their participation and provided written informed consent.

### 2.1 Participant

For the recruitment of subjects, we limited the participation of students majoring in music and psychology, because this study is about the recognition of emotions stimulated by music. We also sent questionnaires to recruited subjects to test their musical perception ability (Seashore, 1923), which is helpful for us to screen the dyslexia. The musical ability of the subjects was tested from three aspects: music appreciation ability, music skill, and music rhythm sense. A total of 91 college students participated in our experiment. According to the scores of the subjects in these three aspects, we selected 84 subjects who scored between 21 and 35 points, which indicated that they had certain music appreciation ability, the overall music level was medium and they had a sense of rhythm, which met our experimental requirements.

In addition, participants were required to be right-handed, have no hearing or visual impairments, be in good health, and have no history of mental illness or bad habits. Prior to the experiment, all subjects signed written informed consent. Participants were not allowed to smoke or consume caffeine 24 h before the experiment. Four subjects were excluded from the final analysis due to a large number of signal artifacts. Therefore, the results analysis was based on a sample of 80 subjects, including 35 males and 45 females.

### 2.2 Experimental materials

**FIGURE 1:**
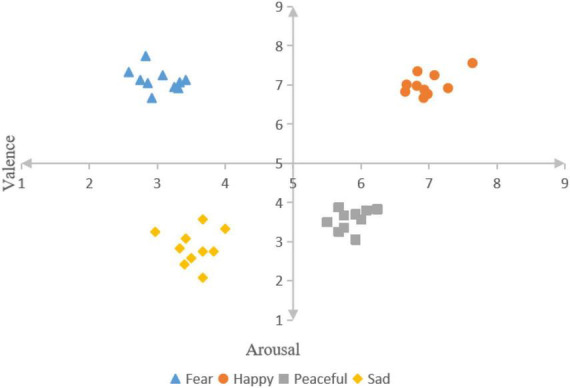
The valence and arousal value of the selected music material.

First of all, music materials were selected from four different emotions (including fear, happiness, calm, and sadness) downloaded from China’s famous NetEase Cloud music platform, and the top 15 songs in each emotion were selected, with a total of 60 songs. These music materials are light music, avoiding the influence of lyrics and language on the subjects. Next, we took the shortest playing time as the standard, cut each piece of material for 20 s, and the sound effect was faded in and out, so that the subjects could better immerse themselves in the music. Finally, 20 non-music major college students aged 18–25 were recruited to score the valence and arousal of the selected music materials on a scale of 1–9 points according to the SAM scale. The mean value and standard deviation of these scores were calculated through statistical analysis. From each emotional stimulus, 10 musical elements that best fit Russell’s two-dimensional emotion model were selected as the final experimental elements. As shown in Figure 1, fear (valence: 3.03 ± 0.29, arousal: 7.12 ± 0.28), happiness (valence: 6.98 ± 0.30, arousal: 7.02 ± 0.28), calm (valence: 5.85 ± 0.22, arousal: 3.56 ± 0.27), and sad (valence: 3.55 ± 0.29, arousal: 2.86 ± 0.45).

### 2.3 Experimental equipment

The experiment was conducted in a temperature-controlled laboratory. The equipment used in this experiment was ActiveTwo 125 acquisition system produced by BIOSEMI Company in Amsterdam, Netherlands, and data acquisition was completed on LabVIEW software. In this study, 128 electrodes were used to record EEG signals at a sampling rate of 1,024 Hz. The headphones used were Sony MDR-EX15LP in-ear headphones, and the volume was controlled at 40% of the computer volume. Subjects performed instructions on a 24 inch screen.

### 2.4 Experimental description

**FIGURE 2:**
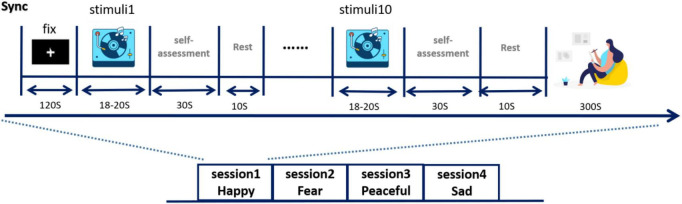
Experimental design process.

Prior to the study, each subject’s scalp was cleaned as required to ensure EEG collection. A cap with 128 electrodes was placed on the subject’s head and the EEG signals were checked and recorded to avoid interference with EEG collection due to improper electrode placement. Then, the experiment protocol and the meaning of the scale used in the experiment were explained to the subjects. When it was confirmed that all subjects understood, the formal experiment began. The experimental process is shown in Figure 2. The experiment consists of three stages:

1. Baseline stage: after subjects understand the experimental scheme, subjects wear headphones and watch the fixation points on the screen. Press the “Start” button as instructed on the screen, then play the music clip of “Forest rain,” and record EEG signals at the same time. This lasted 2 min and put the subject’s mood in a neutral state [the origin in Russell’s model (Russell, 1980)].
2. Musical stimulation and self-evaluation stage: in this stage, 10 pieces of musical stimulation with specific emotions were played to the subjects, each lasting 18–20 s. Immediately after each musical stimulus segment, the subjects filled out the SAM scale based on their true feelings, then rested for 10 s, then moved on to the next musical stimulus segment, rated it, and rested until the 10 pieces of music were played. The subjects then sat quietly for 5 min, waiting for their mood to recover.
3. For the next set of experiments, play another mood specific music, repeat steps (1) and (2). Music for each specific mood for a session. The four sessions are played in the order of fear, happiness, calm, and sadness (Hsu et al., 2017).

Limiting the time for participants to rate the music material to 30 s is intended to make the participants’ rating based on their immediate feelings after listening to the music, rather than overthinking it. The subjects used SAM-9 sub-scale (Zeigler-Hill and Shackelford, 2020) to score the emotional valence and arousal after listening to the music. The valence was a measure of how pleasant or unpleasant the participants felt after listening to the music, with a score of nine being particularly pleasant and a score of one being angry or angry. Arousal refers to the degree to which the subject’s emotions are aroused. The more excited and excited the subject is, the closer the score is to 9, while the score is close to 1 if the emotion is very calm.

## 3 Proposed method

In this section, we first introduce our proposed framework for EEG-based emotion recognition, and then we overview into the details of our EEG preprocessing techniques. Lastly, we provide a detailed description of the construction of the proposed CSBN. SWU-M dataset was used in the experiment.

### 3.1 Proposed CSBN framework

**FIGURE 3:**
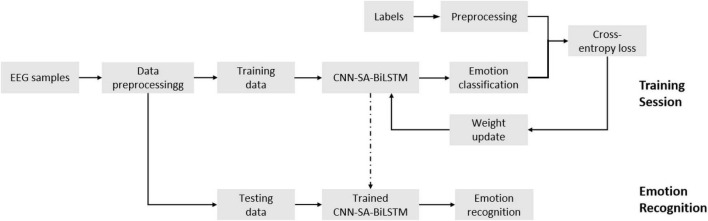
The overall framework of emotion recognition based on CSBN network.

Generally speaking, the original EEG signal contains a lot of unnecessary information, such as electromyography, electrooculogram and environmental noise, which will cause interference to the subsequent emotion recognition. Therefore, in most studies, the raw EEG signals were preprocessed first, and then relevant features were extracted for further analysis. (Li et al., 2016; Alarcao and Fonseca, 2017). The proposed CSBN is a data-driven approach that effectively captures both global and temporal information as emotional features. These features are subsequently classified using the softmax function. Therefore, the accuracy of emotion recognition based on EEG is improved (Figure 3). Firstly, the collected EEG samples are pretreated and their differential entropy (DE) features are extracted. Then, the extracted samples are divided into training samples and test samples. Next, we use training samples to train the proposed CSBN model, carry out cross entropy loss, and use Adam optimizer to update network parameters (Kinga and Adam, 2015). In the final step the trained model is utilized to classify the emotional states of the test samples, and the accuracy of the classification is considered as the ultimate result.

### 3.2 Data preprocessing

During the collection of EEG signals, there are many unnecessary signals, namely noise, including electrocardiogram, electroophthalmic, electromyographic, electrocutaneous, and head movement, which will interfere with subsequent EEG signal analysis. In this experiment, the EEGLAB toolbox in MATLAB R2020b was used to process the original EEG signals collected and remove artifacts, the processing involved artifact removal to ensure signal stability and preservation of relevant data segments. Additionally, a band-pass filter (1–45 Hz) was applied to the continuous EEG data to eliminate linear trends. Then, the signal was downsampled, and the sampling frequency of 128 Hz was realized on the premise of preserving the valid data. For the processing of electroocular and cephalic artifacts, independent component analysis (ICA) in EEGLAB was used to decompose EEG signals into 60 independent components. After artifacts were processed by an automatic toolbox, artifacts were removed by visual inspection, and relatively clean EEG data was obtained. In addition, we chose the first 2 s of stimulation as a time window for baseline correction. The EEG signal was then segmented using a 2 s non-overlapping Hanning window, with each segment being 128 × 256. Finally, a Butterworth filter of order 3 and type bandpass was applied to decompose the EEG into four frequency bands: theta (4–8 Hz), alpha (8–13 Hz), beta (13–30 Hz), and gamma (30–45 Hz).

For label processing, this study considered two classification strategies, such as LV and HV, LA and HA. Based on subjects’ scores of 1–9 on the valence and arousal of musical stimulus fragments, LV/HV and LA/HA class labels were assigned as 0 and 1, and the threshold was set as 5. That is, for LV/HV and LA/HA classification tasks, those with scores lower than 5 were assigned as label 0, and those with scores higher than 5 were assigned as label 1.

After removing the samples inconsistent with the labeled emotions, there were 9,872 samples of the four emotions, including 2,613 samples of fear emotion, 2,709 samples of happiness emotion, 2,384 samples of calm emotion, and 2,166 samples of sadness emotion.

### 3.3 Construction of proposed CSBN

**FIGURE 4:**
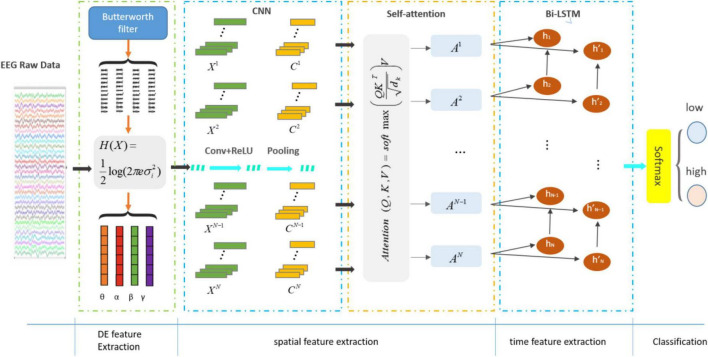
The structure diagram based on CSBN network.

The proposed structure of CSBN is shown in Figure 4. The module consists of the following sections:

1. Differential entropy feature extraction module: the processed EEG signals were segmented into four distinct frequency bands (theta, alpha, beta, and gamma), each representing various states of brain activity. This segmentation was achieved through a third-order Butterworth filter, and subsequently, DE features were extracted from each band.
2. Deep feature extraction module: extracted the local features of EEG signals using CNN and outputted a one-dimensional vector through the last layer of CNN. On this basis, attention mechanism was added to capture the long-distance dependence of the signal and extract more distinguishing global information.
3. Time feature extraction module: the obtained vector was utilized as input for a Bi-LSTM, enabling the prediction of emotional states by incorporating both past and future information within the temporal sequence.
4. Softmax module: softmax classifier mapped all emotion-related information into class labels to obtain emotion classification.

#### 3.3.1 DE feature extraction module

Differential entropy was used as a feature in this study. The concept of DE was equivalent to the concept of entropy of continuous distribution in Shannon’s (1948) original paper, and DE could be used to measure the complexity of continuous signals. DE measured relative uncertainty (Michalowicz et al., 2013), or changes in uncertainty, rather than calculated an absolute measure of uncertainty. DE features could capture information comprehensively, and DE features were calculated based on the probability distribution of EEG signals, which could fully capture the statistical characteristics of the signal. This provided a more comprehensive view of the information than other methods such as power spectral density (PSD) or autoregressive (AR) models, which focused primarily on frequency or time series properties. Compared with the original signal, which contained a large number of noise components and a large number of time series data points, the difference entropy feature could effectively reduce the data dimension, reduce the complexity of data processing and analysis, reduce the impact of noise on feature extraction, and improve the accuracy of emotion recognition. Duan et al. (2013) introduced DE as a feature into EEG emotion recognition for the first time, and the results showed that DE was more suitable for emotion recognition than the traditional feature energy spectrum (ES). Research has demonstrated that DE exhibits discriminative capabilities in discerning between balanced low-frequency and high-frequency EEG patterns. Moreover, the DE features extracted from EEG data offer reliable and precise information, contributing to the stable and accurate classification of emotions (Zheng et al., 2014). Previous studies have proved that DE feature is the best feature extraction method in frequency domain deep learning classification (Song et al., 2018; Liu et al., 2020).

Considering the continuous time random variable *X*, *p*_*X*_(*x*) is the probability density function (PDF) of *X*, then the DE of *X* is defined as the Equation 1:


(1) $$h_{X}=-\int_{S}p_{X}\,\left(x\right)\,l\,o\,g\,\left(p_{X}\,\left(x\right)\right)\,dx$$

Where *S* = {*x*|*p*_*X*_(*x*) > 0} is the support set of *X*, since the random variable conforms to the Gaussian distribution *N*(*μ,σ*^2^), the DE calculation formula of this variable *X* is Equation 2:


(2) $$p\,\left(x\right)=\frac{1}{\sqrt{2\,\pi \,\sigma }}\,e^{-\frac{x^{2}+\mu^{2}}{2\,\sigma^{2}}\,\text{cosh}\,\left(\frac{\mu \,x}{\sigma^{2}}\right)}$$

Then DE can be calculated as Equations 3, 4:


(3) $$h_{X}=\int_{-\infty }^{+\infty }p\,\left(x\right)\,l\,n\,\left(p\,\left(x\right)\right)\,dx$$

(4) $$h_{X}=\frac{1}{2}\,l\,o\,g_{2}\,\left(2\,\pi \,e\,\sigma^{2}\right)+L\,\left(\frac{\mu }{\sigma }\right)$$

Where *L*(⋅) is a function of *μ/σ* with values ranging from 0 to 1 (*ln*2), e is Euler’s constant, and σ is the standard deviation of *x*.

The DE features are computed for all EEG samples across four frequency bands, resulting in the formation of a DE feature matrix. The DE feature matrix can be represented as follows Equation 5:


(5) $${\text{X}}_{\text{d}}^{\text{P}}={\text{[x}}_{\text{d}}^{\text{P}}\left(1\right),{\text{x}}_{\text{d}}^{\text{P}}\left(2\right),\ldots {\text{x}}_{\text{d}}^{\text{P}}\left(n\right)]$$

Where P is the number of electrode channels, d is the number of frequency bands, and N is the number of EEG samples.

#### 3.3.2 Deep feature extraction module

Convolution neural network was extensively employed in various domains such as signal processing, face recognition and other fields. CNN had three key characteristics: local receptive fields, weight sharing, and downsampling, which could effectively improve network performance (Zhang et al., 2020). The high accuracy of the recognition task is mainly due to its ability to learn local nonlinear features through convolution and nonlinear activation functions (LeCun et al., 2015). As shown in Figure 4, one-dimensional tensors after DE feature extraction are input into CNN to learn deep features.

Convolution neural network consisted of three main layers, namely, convolution, pooling, and full connection layer. There were seven convolution layers in total, one maximum pooling layer and two average pooling layers. The first layer was the input layer, which inputted the DE features of the four rhythms after feature extraction. In this study, maximum and average pooling layers were used, respectively. The maximum pooling layer selected only the maximum value in each feature graph, while the average pooling layer selected the average value in each feature graph. This process effectively decreased the model’s training parameters and enhanced the efficiency of the training procedure. A batch normalization layer (BN) was incorporated after the one-dimensional convolution layer to accelerate the model’s convergence during training, improve its stability, and effectively contribute to regularization, thus mitigating overfitting. Then the spatial Dropout layer was used. Unlike the Dropout layer, the Spatial Dropout layer randomly zeroed out some regions, reducing the interdependence between elements and thus further reducing the risk of overfitting. Thus, the *i*-th feature *C*_*i*_(*i* = 1,2,…*N*) was obtained from the *i*-th DE feature *X_i_* by convolution and activation operations. The model parameters were shown in Table 1.

**TABLE 1:** Size of filters and steps recommended for suggested network.

Stage	Stage setting	Output
Convolution-1	32, strides = ‘2, activation = ’“Relu”	32, 256
Convolution-2	32, strides = ‘2, activation = ’“Relu”	32, 126
Pool_1	2, MaxPool	32, 63
BN_1	BatchNormalization	32, 63
Drop_1	Dropout1D	32, 63
Convolution-3	64, strides = ‘2, activation = ’“Relu”	64, 30
Convolution-4	64, strides = ‘2, activation = ’“Relu”	64, 13
Pool_2	2, AvgPool	64, 6
BN_2	BatchNormalization	64, 6
Drop_2	Dropout1D	64, 6
Convolution-5	128, strides = ‘1, activation = ’“Relu”	128, 4
Convolution-6	128, strides = ‘1, activation = ’“Relu”	128, 3
Pool_3	2, AvgPool	128, 2
BN_3	BatchNormalization	128, 2
Drop_3	Dropout1D	128, 2
Convolution-7	64, strides = ‘1, padding = ’1, activation = ’“Relu”	64, 2
BN_4	BatchNormalization	64, 2

In order to improve the expressiveness of the model and solve the problem of gradient disappearance, residual connections were added to the CNN network. Different from the general additive residuals join, the output of the main path was multiplied with the residuals, passing the information and residuals through element multiplication, expressed as *x*_*l* + 1_ = *x*_*l*_⋅*F*(*x*_*l*_,*W*_*l*_), where *F*(*x*_*l*_,*W*_*l*_) was the residuals part, consisting of two convolution layers. Because *x_l_* and *x*_*l+1*_ dimensions did not match, a 1 * 1 dimension adjustment convolution was required, $h\,\left(x_{l}\right)=W_{l}^{\prime }\,x$, including $W_{l}^{\prime }$ being a 1 * 1 convolution. Finally, it was expressed as *x*_*l* + 1_ = *h*(*x*_*l*_)⋅*F*(*x*_*l*_,*W*_*l*_). The advantage was that it emphasized the complementarity between features and allowed the network to learn the relative weights between features. This connection made the network more flexible, alleviated the problem of disappearing gradients, and helped capture nonlinear relationships in EEG.

However, in CNN, convolution operation was limited to local receptive field, and global dependence could not be directly captured. By learning the weight relationship between each position and other positions, the self-attention mechanism could establish dependencies on a global scale and better capture long-distance context information. Moreover, the self-attention mechanism had better adaptability. Self-attention was not limited by the fixed convolution kernel size and stride length, and could learn the weight relationship adaptively according to different tasks and data. This adaptability made the self-attention mechanism more flexible and generalizing and could perform well on a variety of different types of data and tasks (Ozdemir et al., 2021). Therefore, we added the self-attention mechanism on the basis of CNN to improve the ability of the model to extract features. The self-attention module computed the attention weights by scaling the dot product attention by generating three linear projections [Key (K), Value (V), and Query (Q)] on the input sequence, where Q, K, and V were obtained by linear changes using the inputs of the self-attention mechanism. The output from the attention mechanism was calculated using Q, K, V, where *Q* = *X* *W^q^*, *K* = *X* *W^k^*, *V* = *X* *W^v^*. *W^q^*, *W^k^*, *W^v^* were learnable parameters. These weights were then mapped to the input sequence. The inputs to scaling dot product attention included the *d_k_* of the query and key and the *d_v_* dimension of the value. In a nutshell, we first calculated the dot product of the query with all the keys, divided by $\sqrt{d_{k}}$ for normalization, and then used Softmax to normalize the value to between 0 and 1. The results of the weighted sum of V and the weight distribution were taken as the output of the attention mechanism. The difference of weight distribution directly affected the transmitted information, and the information transmitted according to different weight models was different, which was the essence of attention mechanism.

**FIGURE 5:**
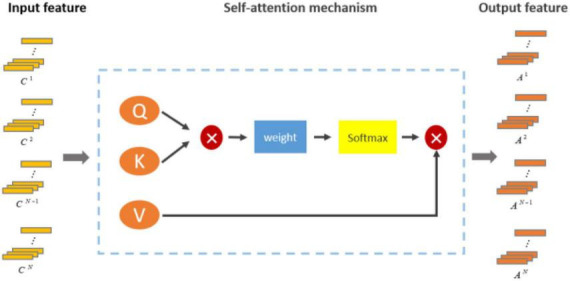
The structure of self-attention mechanism.

The structure of self-attention is shown in the Figure 5. Here, the *i*-th encoded representation after self-attention is {*A*_*i*_|*A*_*i*_ = *Attention*(*C*_*i*_),*i* = 1…..*n*}. Where A is: $A=A\,t\,t\,e\,n\,t\,i\,o\,n\,\left(Q,K,V\right)=s\,o\,f\,t\,m\,a\,x\,\left(\frac{Q\,K^{T}}{\sqrt{d_{k}}}\right)\,V$.

#### 3.3.3 Time feature extraction module

The right side of the structure diagram shows the temporal feature extraction module (Figure 4). RNN was a recursive neural network that inputted sequential data and recurred according to the evolutionary direction of the sequence, and was connected by chains composed of all recursive units (Graves et al., 2013). LSTM was a variant of RNN, whose core state was cell state and gate structure, which could solve the dependence problem that RNN could not handle long distance.

**FIGURE 6:**
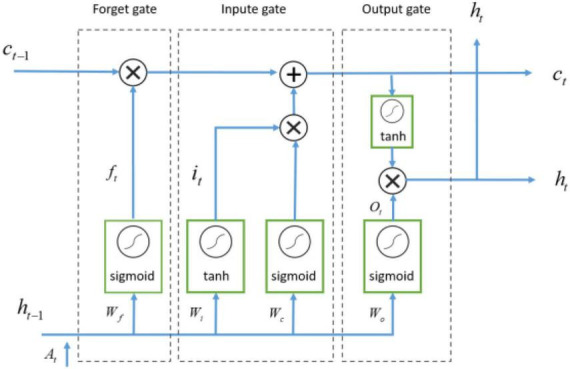
The structure of LSTM network.

Long short-term memory was similar to RNN in main structure. Its main improvement was that three gating structures were added in hidden layer h, namely forgetting gate, input gate and output gate. Data flow was controlled by sigmoid and tanh activation functions. The forget gate discarded some of the past information, while the input gate remembered certain present information, and then these pieces of information were combined and passed through the output gate (Goldstein et al., 2019). Therefore, LSTM network could extract temporal features. As shown in Figure 6.

The LSTM cell took three inputs, namely, the current input at time *t*, denoted as *X_t_*, the previous output at time *t*-1, denoted as *C*_*t*–1_, and the previous hidden state at time *t*-1, denoted as *h*_*t*–1_. Then the LSTM unit produced two outputs: the current output at time *t*, denoted as *C_t_*, the hidden state at time *t* was denoted as *h_t_*, representing the *t*-th time feature extracted from the LSTM. LSTM is calculated as Equations 6–10:


(6) $$f_{t}=\sigma \,\left(W_{f}\cdot \left[h_{t-1},a_{t}\right]+b_{f}\right)$$

(7) $$i_{t}=\sigma \,\left(W_{i}\cdot \left[h_{t-1},a_{t}\right]+b_{i}\right)$$

(8) $$O_{t}=\sigma \,\left(W_{o}\cdot \left[h_{t-1},a_{t}\right]+b_{o}\right)$$

(9) $$c_{t}=f_{t}\times c_{t-1}+i_{t}\times t\,a\,n\,h\,\left(W_{c}\cdot \left[h_{t-1},a_{t}\right]+b_{c}\right)$$

(10) $$h_{t}=O_{t}\times t\,a\,n\,h\,\left(c_{t}\right)$$

Where *A_t_* is the output of time feature extraction module at time*t*, *W_f_*, *W_i_*, *W_o_* and *W_c_* are respectively the weights of forgetting gate, input gate, output gate and cell state. *b_f_*, *b_i_*, *b_o_*, *b_c_* are the bias of forgetting gate, input gate, output gate, and cell state, respectively.

**FIGURE 7:**
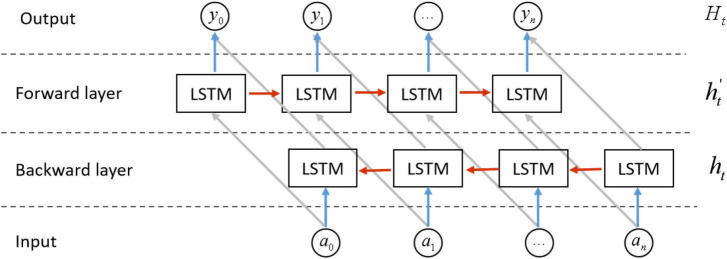
The structure of BiLSTM network.

Although LSTM had been improved significantly compared with RNN, it could solve the problem of gradient vanishing and gradient explosion to a certain extent. LSTM networks, while effective in capturing sequential dependencies, had a limitation in encoding information solely in a forward manner. On the other hand, Bi-LSTM networks were capable of capturing semantic dependencies in both forward and backward directions, resulting in enhanced ability to model bidirectional relationships. The Bi-LSTM neural network architecture consists of two separate and independent LSTM layers, as shown in Figure 7, the input sequence is respectively in positive order and reverse order to the two LSTM neural networks for feature extraction, the feature vectors output by the two LSTM are spliced to form a new vector as the final output vector.

The design of the Bi-LSTM model aimed to enable the feature data obtained at time t to incorporate both past and future information simultaneously. The experimental results indicated that this model architecture outperformed a single LSTM model in terms of feature extraction for time series data (Graves et al., 2005). A noteworthy aspect was the independent nature of the two LSTM neural network parameters within the Bi-LSTM model. This independence enabled effective collaboration between the parameters, ensuring the preservation of previous information and thereby enhancing the model’s capacity to capture valuable insights from EEG time series data.

The fundamental idea of BiLSTM was to encode each sequence by employing two independent hidden states. These states were specifically designed to capture past and future data individually. The model subsequently interlinked these two distinct hidden states, thereby creating a comprehensive view of the sequence. The final output was {*H*_*i*_|*H*_*i*_ = *BiLSTM*(*A*_*i*_),*i* = 1…*n*}.

In this study, the output features after the attentional mechanism layer were input into the BiLSTM layer, which consisted of three bidirectional BiLSTM layers, with 128 neurons in the first layer and 64 neurons each in the second and third layers.

#### 3.3.4 Softmax module

In the concluding segment of the proposed CSBN model, we employed the softmax layer as the classifier, thereby enabling effective categorization. As shown in Equation 11:


(11) Y=s⁢o⁢f⁢t⁢m⁢a⁢x⁢(W⁢H+b)

where *Y* = {*y*_1_, *y*_2_, …, *y*_*n*_}, *y*_*i*_(*i* = 1…*n*) denotes the forecasted probability of the *i*-th EEG sample, while *W* and *b* represent the weight and bias parameters of the softmax function, respectively.

In summary, we designed a framework for extracting temporal and spatial features from EEG signals and classifying emotions. DE module was used to extract DE features from EEG signals, advanced features of EEG signal were extracted by CNN, and then self-attention mechanism was used to self-allocate different weights and extract global information. In addition, we used BiLSTM to dig the temporal characteristics from EEG samples, and finally carried out emotion recognition on the obtained temporal and spatial characteristics.

## 4 Results

In this part, we designed an ablation experiment to verify the effectiveness of the proposed model, and evaluated the classification performance of the combination of each part of the model through 10-fold cross-validation. At the same time, because most EEG algorithms focus on the whole band, the information between single band and frequency band combination is ignored. Therefore, we discuss the influence of θ, α, β, and γ bands on music emotion recognition and the influence of multi-band combination on music emotion recognition.

### 4.1 Experimental result

To verify the effectiveness of this method, an ablation experiment was designed on the SWU-M dataset, which included CNN, BiLSTM, and CNN + BiLSTM models. The details of these models are shown in Table 2. The ability of emotion recognition of a single module is verified by CNN and BiLSTM. CNN + BILSTM is a combination of CNN and BiLSTM module which is used to verify the ability of baseline model to extract temporal features. The results of ablation recognition are shown in Table 2. The experimental results show that CSBN module has the highest classification accuracy of valence and arousal, and the recognition accuracy of valence and arousal is 93.45% and 96.36%, respectively, after 10 cross-validations. Compared with CNN and BiLSTM, the features extracted by CNN and BiLSTM are too single, so the recognition effect is not good. However, CNN-BiLSTM only focuses on local information and cannot directly capture global dependencies, so it does not have good recognition performance. CSBN showed good emotion recognition ability because of its ability to extract temporal features and its ability to use global information.

**TABLE 2:** Ablation experimental model and the average accuracy of the ablation experimental model under the 10-fold cross-validation on valence and arousal classification tasks.

	CNN	Self-attention	LSTM network	Valence	Arousal
CNN	√	×	×	88.23	89.84
BiLSTM	×	×	√	87.29	89.17
CNN-BiLSTM	√	×	√	91.04	91.96
CSBN	√	√	√	93.45	96.36

In addition, the proposed method is compared with two deep learning methods, RNN and ResNet. At the same time, Random Forest Algorithm (RF) and Decision Tree (DT) two traditional machine learning methods are compared. As shown in Table 3. All methods underwent the same preprocessing as CSBN. Experimental results show that compared with the traditional machine learning method DT, the accuracy of the proposed method is improved by about 20%. Compared with the RF method, the accuracy of this method is improved by about 15%. Compared with the two deep learning methods (RNN and ResNet), the CSBN method proposed in this study improves by about 5%, showing superior emotion classification performance.

**TABLE 3:** The average accuracy of 10-fold cross-validation for different methods on valence and arousal classification tasks.

	Valence	Arousal
DT	74.67	78.31
RF	79.48	83.99
RNN	87.77	89.84
ResNet	88.54	91.28
CSBN	93.45	96.36

**FIGURE 8:**
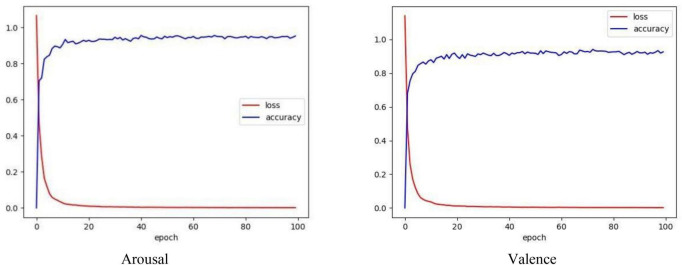
Loss and accuracy graph by using CSBN model with 10-fold cross validation.

Figure 8 depicts the loss and accuracy curves obtained from a 10-fold cross-validation using the CSBN model. Loss refers to the disparity between the model’s predicted value and the actual value. The loss function used in our model is the classification cross entropy. Accuracy is one of the indicators to evaluate the performance of our model. It is clear from the figure above that under 10-fold cross validation, the model minimizes the loss to 7.63% at 100 epochs, and our model achieves high accuracy in the valence and arousal tasks, respectively.

To corroborate the model’s performance more comprehensively, Table 4 also summarizes the experimental results of the CSBN model on the valence and arousal labels of average EEG emotion recognition Accuracy, Precision, F1-score, and AUC rate, which fully indicate that the proposed model has good stability and classification performance.

**TABLE 4:** The average experimental results of 10-fold cross-validation for CSBN on valence and arousal classification tasks.

	Valence	Arousal
Accuracy (Acc)	93.45	96.36
F1-score	92.72	96.61
AUC	95.36	97.81
Precision (Pre)	93.24	96.65

### 4.2 The influence of single band on music emotion recognition

CSBN was used to identify musical emotion in each rhythm of music-induced EEG signals.

As shown in Table 5, the experimental results show that for the classification of arousal, the Accuracy of CSBN network for θ-band EEG is 84.38%, the Precision is 84.80%, the AUC is 83.99%, and the accuracy of F1-score is 84.51%. Similarly, CSBN network is used to evaluate the accuracy, sensitivity, AUC, and F1 scores of α, β, and γ bands, and it is found that the Accuracy, Precision, AUC, and F1 scores of LA and HA emotion classification methods are all above 80%. Similarly, for valence classification, the accuracy, sensitivity, AUC, and F1-score of CSBN network single band are all above 80%. At the same time, it can also be found that whether it is valence or arousal classification, the classification accuracy of α band is the highest in single band emotion recognition, indicating that α band has stronger emotion perception ability, which verifies previous studies (Iwaki et al., 1997) that musical stimuli usually cause an increase in α band activity.

**TABLE 5:** The average experimental results of 10-fold cross-validation for CSBN on single-band valence and arousal classification tasks.

Classification task	Rhythm	Acc	Pre	AUC	F1-score
Arousal	θ	84.38	84.80	83.99	84.51
	α	87.50	88.10	87.50	87.45
	β	82.81	83.65	82.81	82.7
	γ	85.94	86.60	85.73	85.97
Valence	θ	81.25	82.59	81.76	81.20
	α	85.94	87.38	87.08	86.13
	β	81.66	83.14	81.68	81.82
	γ	84.38	85.63	84.38	84.25

### 4.3 The influence of multi-band on music emotion recognition

As shown in Table 6, in the study of the influence of multi-band combination on music emotion recognition, the experimental results show that the recognition accuracy of the combination of α, θ, β, and γ bands, (θ, α), (α, β), and (α, γ) bands is higher than that of the combination of other bands (θ, β) and (θ, γ), among which the recognition accuracy of the combination of (α, γ) band is the highest.

**TABLE 6:** The average experimental results of 10-fold cross-validation for CSBN on combined-band valence and arousal classification tasks.

Classification task	Rhythm	Acc	Pre	AUC	F1-score
Arousal	(θ, α)	92.41	93.27	92.42	92.16
	(θ, β)	88.94	90.28	90.04	89.12
	(θ, γ)	92.18	92.24	92.25	92.19
	(α, β)	91.29	92.22	92.19	92.18
	(α, γ)	93.75	93.95	93.99	93.76
	(β, γ)	92.07	92.62	92.56	92.20
	(θ, α, β)	93.63	93.27	93.20	93.61
	(θ, α, γ)	95.31	95.71	95.31	95.30
	(θ, β, γ)	92.81	92.65	94.60	92.45
	(α, β, γ)	95.15	95.22	96.23	94.86
Valence	(θ, α)	87.39	87.23	86.94	86.06
	(θ, β)	82.50	82.81	82.64	82.79
	(θ, γ)	86.04	86.82	85.53	85.85
	(α, β)	89.06	89.15	89.04	89.09
	(α, γ)	90.63	90.80	90.71	90.63
	(β, γ)	89.42	89.68	90.31	88.95
	(θ, α, β)	90.67	90.52	91.47	90.59
	(θ, α, γ)	92.62	92.36	93.39	92.45
	(θ, β, γ)	91.71	91.62	91.60	91.59
	(α, β, γ)	91.35	91.18	92.16	92.26

In the combination of three bands, the combination of (α, β, and γ) has better recognition rate. For the classification of arousal, the Accuracy, Precision, AUC, and F1-score are 95.31%, 95.71%, 95.31%, and 0.953, respectively. For the classification of valence, the Accuracy, Precision, AUC, and F1-score are 92.62%, 92.36%, 93.39%, and 0.9245, respectively. At the same time, the above table can also be found. The recognition rate of the combination of three frequency bands is better than that of two frequency bands, because the three frequency bands contain more complementary information, which is conducive to emotion classification. Any combination of bands containing alpha will achieve relatively high accuracy, which is consistent with neuroscience studies showing that rhythmic stimuli as well as loud and calm music increase alpha-band activity (Rogers and Walter, 1981).

## 5 Discussion

To verify the effectiveness and generalization of the proposed algorithm, we conducted experiments on a widely used DEAP dataset (Koelstra et al., 2011). Then we compared the proposed method with several published studies.

### 5.1 Comparison with other datasets

DEAP dataset was a multi-channel physiological dataset used for studying emotional states. This dataset was publicly available and free to access.

The dataset consisted of 32 EEG channel signals and 8 peripheral physiological signals recorded by 32 subjects while watching 40 music videos. In this study, only EEG was used for emotion recognition, other signals were abandoned. The data were standardized in this study. First, the EEG signal of 512 Hz was down sampled to 128 Hz, then band-pass filtering was performed at 1–45 Hz, and ICA was used to remove the interference of EEG signal. Each participant in the study watched 40 emotional music videos, each lasting 60 s. After viewing the videos, the participants rated valence, arousal, liking, and dominance using a 9-point scale. In this experiment, only valence and arousal were used as emotional evaluation criteria, and 5 was taken as the scoring threshold. Labels with scores higher than 5 were labeled as 1 (positive valence), while those with scores lower than 5 were labeled as 0 (negative valence).

Table 7 is a comparative analysis of the EEG data sets, including the number of participants, the number of EEG devices with different channel numbers, the type and duration of stimulation, and the categories of emotions awakened. DEAP data was processed by the same sliding time window as SWU-M and divided into 2 s time segments.

**TABLE 7:** Analysis of EEG data sets.

Data (year)	No. of subjects	No. of electrodes	Stimuli duration(s)	Stimuli	No. of emotions	Emotions
DEAP (2011)	32	32	60	Video	4	High/low valence High/low arousal
SWU-M (2022)	80	128	20	Music	4	High/low valence High/low arousal

As can be seen from Table 8, under 10-fold cross-validation, the highest accuracy of binary classification based on CSBN model on DEAP data set is arousal: 95.31%, valence: 93.64%. The average accuracy is arousal: 93.17%, valence: 92.90%. On the SWU-M dataset, the highest accuracy of binary classification is arousal: 97.20%, valence: 94.79%. The average accuracy is arousal: 96.36%, valence: 93.45%.

**TABLE 8:** Performance comparison of CSBN model on other EEG datasets under 10-fold cross-validation.

Dataset	Valence		Arousal	
	Max	Mean	Max	Mean
DEAP	93.64	92.90	95.31	93.17
SWU-M	94.79	93.45	97.20	96.36

Furthermore, we compared the CSBN method with two deep learning approaches, RNN and ResNet, on the DEAP dataset. Simultaneously, we conducted comparisons with two machine learning methods (RF and DT). As shown in Table 9, the Accuracy, Precision, AUC, and F1-score of various methods on the DEAP data set were calculated respectively. These results showed that the CSBN model could also achieve better accuracy on other publicly available benchmark datasets, which validated the validity and generalizability of our approach.

**TABLE 9:** Performance comparison of different methods under 10-fold cross-validation on DEAP data sets.

	Methods	Acc	Pre	AUC	F1-score
Valence	DT	72.48	71.22	72.31	71.29
	RF	75.93	73.79	74.61	73.25
	RNN	82.81	83.03	81.49	82.89
	ResNet	89.06	89.06	88.82	89.04
	CSBN	92.90	92.75	92.90	92.59
Arousal	DT	73.72	73.72	72.61	73.41
	RF	78.44	76.17	75.53	76.40
	RNN	85.94	86.01	84.51	85.78
	ResNet	90.63	90.96	90.77	90.70
	CSBN	93.17	92.57	92.96	92.73

Compared with the visual and auditory dual stimuli of the DEAP dataset, our dataset was collected only under auditory stimuli. As the number of sensory stimuli increased, the subjects’ emotions were also more strongly aroused. However, the results showed that our model achieved good accuracy even under a single auditory stimulus, which proved the superiority of our method.

Therefore, we had reason to believe that our classification results were also reliable on other data sets, which also proved the generalization of our model. The experimental results showed that our model achieved better results than other models and also achieved good results on publicly accessible EEG datasets. These findings indicated the universality of our approach and its potential to address complex problems.

### 5.2 Comparison with existing classification methods

Finally, we compare the proposed method with several published studies, as shown in Table 10. Compared with machine learning algorithm (Liu et al., 2017; Gao et al., 2020; Zhou et al., 2021), the accuracy has been greatly improved. Compared with a single CNN or LSTM (Alhagry et al., 2017; Zhan et al., 2019), the proposed approach in this study represents a significant improvement. The results demonstrate that global features have a substantial impact on the accuracy of emotion recognition, and information about the future and past is also important in the dynamic characteristics of time, while single information has a poor influence on the accuracy of emotion recognition. However, CNN-LSTM and CNN-HMMS (Zhang et al., 2020; Zhong et al., 2020; Jiang et al., 2021a) do not learn future emotional states in the EEG time series, so its accuracy is still poor. Although LSTM-attention (Du et al., 2020) learns the temporal characteristics of EEG signals, the lack of processing global features results in poor classification performance. DE-CNN-BiLSTM (Cui et al., 2022) fully considers the complexity of the brain, but this method do not consider the use of attention mechanism to redistribute the weight of key information in EEG signals. CSBN uses CNN to capture advanced features in EEG, and then uses self-attention mechanism to reassign the weight of these information. Finally, BiLSTM is used to fully learn the past and future key emotional information in EEG signals, so that the network has better recognition ability.

**TABLE 10:** Comparison with existing classification methods.

References	Datasets	Stimuli	Inputs	Classifier	Accuracy	
					Valence	Arousal
Alhagry et al., 2017	DEAP	Audio-visual (music and video clips)	Raw EEG signals	LSTM	85.45%	85.65%
Zhan et al., 2019	DEAP	Audio-visual (music and video clips)	PSD	CNN	82.95%	84.07%
Zhong et al., 2020	DEAP	Audio-visual (music and video clips)	MSE	CNN-HMMS	83.09%	79.77%
Gao et al., 2020	DEAP	Audio-visual (music and video clips)	PSD	SVM-RBF	62.49%	62.17%
Zhang et al., 2020	DEAP	Audio-visual (music and video clips)	Raw EEG signals	CNN + LSTM	90.12%	94.17%
Du et al., 2020	DEAP	Audio-visual (music and video clips)	DE	LSTM + attention	90.91%	90.87%
Liu et al., 2017	Self-acquisition dataset	Audio-visual (film clips)	PSD, ASM	SVM-RBF	Positive 86.43% Negative 65.09%	–
Zhou et al., 2021	Self-acquisition dataset	Audio-visual (music and film clips)	PSD, DE	Random forest (RF)	78.75%	73.98%
Jiang et al., 2021a	Self-acquisition dataset	Audio-visual (music and video clips)	Normalization EEG signals	CNN + LSTM	Avg 93.13%	
Cui et al., 2022	DEAP/SEED	Audio-visual (music and video clips)	DE	DE-CNN-BILSTM	94.02%	94.86%
Proposed method	Self-acquisition dataset	Audio (music)	DE	CSBN	93.45%	96.36%

## 6 Conclusion and future work

In this study, we proposed a CSBN method, which could make better use of EEG to classify music-induced emotions, and fully considered the characteristics of EEG information. Firstly, the collected original data was preprocessed, its DE features were extracted, and then input into the CSBN model. On the SWU-M dataset, the average accuracy of arousal and valence was 96.36% and 93.45%, respectively. To further verify the validity of this model, we used the DEAP dataset for experiments. The average accuracy of valence on the DEAP dataset was 92.90% and the average accuracy of arousal was 93.17%, indicating that the model had good robustness and generalization ability. At the same time, the effects of musical stimulation on different electrical bands of the brain were also studied, and the study showed that music increased activity in the alpha band. Compared with other music emotion recognition work, the methodology proposed in this study demonstrated superior classification performance. This held significant implications for future exploration within the field of emotion recognition systems based on brain-computer interfaces.

This study also has the following deficiencies:

1) Since this study only focused on the classification of valence and arousal, the model will be optimized in the future and further applied to the multi-classification emotion recognition task of multi-channel EEG.
2) Since the training of this model was supervised and required the collection of a large number of labeled EEG signals, future work will incorporate transfer learning technology to decrease reliance on labeled signal data.
3) Since this study classified offline emotions stimulated by music, future work will focus on online emotion recognition, quickly processing and analyzing real-time input data, and more accurately understanding and interpreting the changing process of emotions according to the flow of information.

## Data availability statement

The original contributions presented in this study are included in the article/supplementary material, and further inquiries can be directed to the YQ.

## Ethics statement

The studies involving humans were approved by the Ethical Review Committee of Southwest University. The studies were conducted in accordance with the local legislation and institutional requirements. The participants provided their written informed consent to participate in this study.

## Author contributions

GL: Funding acquisition, Investigation, Methodology, Project administration, Resources, Supervision, Validation, Writing - review & editing. YQ: Conceptualization, Data curation, Formal analysis, Investigation, Methodology, Software, Validation, Writing - original draft, Writing - review & editing. JM: Investigation, Methodology, Writing - review & editing. JX: Supervision, Validation, Writing - review & editing. BH: Formal analysis, Supervision, Validation, Writing - review & editing.
